# Reliable Sarcoidosis Detection Using Chest X-rays with EfficientNets and Stain-Normalization Techniques

**DOI:** 10.3390/s22103846

**Published:** 2022-05-19

**Authors:** Nadiah Baghdadi, Ahmed S. Maklad, Amer Malki, Mohanad A. Deif

**Affiliations:** 1 Nursing Management and Education Department, College of Nursing, Princess Nourah Bint Abdulrahman University, P.O. Box 84428, Riyadh 11671, Saudi Arabia; nabaghdadi@pnu.edu.sa; 2 Computer Science Department, College of Computer Science and Engineering in Yanbu, Taibah University, Medina 42353, Saudi Arabia; asamalki@taibahu.edu.sa; 3 Information Systems Department, Faculty of Computers and Artificial Intelligence, Beni-Suef University, Beni-Suif 62521, Egypt; 4 Department of Bioelectronics, Modern University of Technology and Information (MTI University), Cairo 12055, Egypt; mohand.deif@eng.mti.edu.eg

**Keywords:** pulmonary sarcoidosis, sarcoidosis detection, tuberculosis, chest X-rays, EfficientNets, stain normalization

## Abstract

Sarcoidosis is frequently misdiagnosed as tuberculosis (TB) and consequently mistreated due to inherent limitations in radiological presentations. Clinically, to distinguish sarcoidosis from TB, physicians usually employ biopsy tissue diagnosis and blood tests; this approach is painful for patients, time-consuming, expensive, and relies on techniques prone to human error. This study proposes a computer-aided diagnosis method to address these issues. This method examines seven EfficientNet designs that were fine-tuned and compared for their abilities to categorize X-ray images into three categories: normal, TB-infected, and sarcoidosis-infected. Furthermore, the effects of stain normalization on performance were investigated using Reinhard’s and Macenko’s conventional stain normalization procedures. This procedure aids in improving diagnostic efficiency and accuracy while cutting diagnostic costs. A database of 231 sarcoidosis-infected, 563 TB-infected, and 1010 normal chest X-ray images was created using public databases and information from several national hospitals. The EfficientNet-B4 model attained accuracy, sensitivity, and precision rates of 98.56%, 98.36%, and 98.67%, respectively, when the training X-ray images were normalized by the Reinhard stain approach, and 97.21%, 96.9%, and 97.11%, respectively, when normalized by Macenko’s approach. Results demonstrate that Reinhard stain normalization can improve the performance of EfficientNet -B4 X-ray image classification. The proposed framework for identifying pulmonary sarcoidosis may prove valuable in clinical use.

## 1. Introduction

Tuberculosis (TB) is an infectious disease and one of the top 10 causes of death worldwide [1,2]. Despite major advances in tuberculosis control methods, such as improved vaccines and novel treatments, there are still difficulties in the development of quick and accurate TB testing procedures [3]. Multidrug-resistant tuberculosis (MDR TB) has emerged as the most difficult disease to treat, and it is spreading fast, demonstrating the pathogen’s adaptability [4]. Around 23% of the world’s population has latent tuberculosis [4,5]. In the developing world, tuberculosis is still a major, life-threatening disease, particularly in countries with high population density and poor sanitation. Tuberculosis elimination has become a major public health concern, and the urgency of the effort has been compounded by the advent of new tuberculosis bacillus strains that are resistant to standard medicines [6]. Extrapulmonary tuberculosis (EPTB) occurs when tuberculosis spreads outside of the lungs. Tuberculosis of the lungs (PTB) and EPTB may coexist. Asymptomatic people account for 15 to 20% of the population [7].

Sarcoidosis is a multisystemic granulomatous disease characterized by lumps in the lungs, skin, or lymph nodes produced by an abnormal inflammatory cell accumulation [8]. Sarcoidosis can often be cured without medication. Sarcoidosis is a clinically similar disease to tuberculosis with an unknown cause [9]. Sarcoidosis and PTB are both granulomatous diseases with comparable clinical-radiological presentations, making differentiation challenging in regions where they occur often [6].

These two diseases were long assumed to be the same because of their similar symptoms and histology. Although tuberculosis can be a side effect of sarcoidosis treatment, the two diseases rarely coexist. Despite this, there have been cases of tuberculosis and sarcoidosis coexisting, with symptoms ranging from pulmonary [10] to extrapulmonary [5] and presenting in a variety of ways. In several studies, MTB deoxyribonucleic acid (DNA) was associated with a large proportion of tissue and bronchoalveolar lavage samples from sarcoidosis patients. There have, however, been reports of deleterious consequences [2,11]. Differentiating tuberculosis and sarcoidosis can be difficult, especially in cases of mediastinal lymphadenopathy, because both diseases have similar clinical presentations and histopathologically identical granulomatous inflammation [12]. Tuberculosis diagnoses are now based mostly on microbiological confirmation, which is only attainable in 50% of cases [9]. As a result, better diagnostic approaches are needed to reduce morbidity as a result of delayed or inefficient treatment [13].

To diagnose and screen for pulmonary tuberculosis and sarcoidosis, chest X-rays (CXR) are widely employed [14,15,16]. Competent clinicians use chest radiography to diagnose tuberculosis and sarcoidosis in clinical practice. This is, however, a lengthy and subjective procedure. Low-resource countries (LRCs) also have a shortage of radiologists, especially in rural areas [17]. As a result, by analyzing chest X-ray images, computer-aided diagnostic tools can play an essential role in mass screening for pulmonary tuberculosis and sarcoidosis. Artificial intelligence-based solutions for a variety of medical applications, such as the identification of tumors, lung nodules, physiological monitoring, breast cancer, pneumonia, and social sensing, have recently been proposed [6,18].

## 2. Related Work

Convolutional neural networks (CNNs) are types of deep machine learning techniques that have shown a lot of promise in image classification and hence have a lot of support from the scientific community [6,19,20]. Deep learning techniques have become popular for diagnosing lung diseases based on chest radiographs since X-ray radiography is a low-cost imaging modality with many data for training machine learning algorithms. Traditional machine learning algorithms were used by several research groups [12,21,22] to distinguish normal patients from those with tuberculosis using CXR images. By adjusting CNN settings, deep machine learning techniques were applied to classify patients with tuberculosis [23,24,25]; using pre-trained models, transfer learning was used to detect patients with tuberculosis [26,27,28]. Hooda et al. [29] presented a deep learning method that properly identified CXR images as tuberculosis or normal cases with 82.09 % accuracy. Evalgelista et al. [26] reported an 88.76% accuracy rate for TB detection from chest X-ray images using CNNs. With an accuracy of 86.82%, Pasa et al. [27] suggested a deep network architecture for tuberculosis monitoring. A method for interactively monitoring TB instances was also mentioned. Nguyen et al. [28] used a DenseNet to categorize normal and TB images from the Montgomery County and Shenzhen datasets [30] and obtained AUC values of 0.82 and 0.94, respectively. Hernandez et al. [13] proposed an automated tuberculosis classification system based on X-ray images that used CNN and archival data (with an accuracy of 86%). Various pre-trained CNN architectures were used to classify chest radiographs into two categories, namely positive or negative for tuberculosis infection, according to Lopes et al. [31]. The system’s accuracy was determined to be 81% using two publicly available chest X-ray databases. Using four CNN models (GoogLeNet, RestNet50, VGG-16, and VGG-19), Meraj et al. [32] examined the accuracy limits for small- and large-scale CNN models in the classification of tuberculosis from chest X-rays. Ahsan et al. [33] presented a pre-trained CNN model for tuberculosis detection that had an 80% sensitivity. With an accuracy rate of 94.89%, Yadav et al. [34] used the transfer learning model to identify tuberculosis. Abbas et al. [35] presented a CNN model to improve the performance of ImageNet pre-trained CNN models and achieved high TB classification accuracy using the Japanese Society of Radiological Technology (JSRT) database. It should be mentioned that transfer learning techniques were also employed to classify TB culture test images. On labeled tuberculosis culture images, Chang et al. [12] employed the transfer learning method and achieved sensitivity and precision rates of 98 and 99%, respectively. Mahalakshmi et al. [11] used an Artificial Neural Network (ANN) to identify tuberculosis and sarcoidosis based on gene expression. Several machine learning algorithms were compared by Chen et al. [36]. They employed Decision Tree, Support Vector Machine, and Naive Bayes to classify TB and sarcoidosis. Kong et al. [37] established tuberculosis and sarcoidosis detection techniques based on Decision Trees.

Based on past literature reviews, a few different approaches to distinguishing between patients with sarcoidosis and those with tuberculosis have been presented. Furthermore, the number of research datasets is limited, making it difficult to employ machine learning models in the real world. To our knowledge, no other research using deep learning to distinguish pulmonary tuberculosis from sarcoidosis using chest X-ray images has been published. Using different deep learning algorithms, adjusting existing algorithms, or integrating several outperforming techniques into an ensemble model can improve classification performance. Because of the increasing availability of computational power, CNNs have become viable. Techniques for segmentation and feature extraction can be designed without expert topic knowledge; however, for a CNN to produce improved results, the dataset must be annotated. These networks can locate and extract critical information for image classification.

This research distinguishes between pulmonary tuberculosis and sarcoidosis using a CNN transfer-learning-based approach. Transfer learning allows pre-trained models to be reused for better diagnostic results. EfficientNet, AlexNet, ResNet50, VGG-16, and Inception V3 are some of the transfer learning models used in ensemble learning. The use of EfficientNets for X-ray image classification, according to the findings of this study, is a simple and basic technique that saves training time while keeping the same accuracy as previously proposed computationally expensive algorithms.

## 3. Research Contribution

The investigations pursued in this research study offer major contributions. To begin, seven EfficientNet models with transfer learning were examined for identifying chest X-ray images. The currently proposed method was successful in identifying and recognizing the overall features of an image. In the second step, the impact of the stain-normalization approach on image classification was evaluated. Finally, the EfficientNet models’ classification performance was compared to that of other CNN models, such as ResNet50, AlexNet, VGG16, and Inception V3. The goal of these comparisons was to see whether fine-tuned EfficientNets can categorize X-ray images as well as existing framework techniques. The paper is organized as follows: Section 1 is an introduction to the clinical differences between sarcoidosis and TB. The literature review in Section 2 delves in depth into prior effective techniques. Section 3 highlights the research contributions of the study. Section 4 offers a background description of training and transfer learning, as well as the methodology and approaches employed in this paper. The results and discussion thereof are found in Section 5. The findings of the study are summarized in Section 6.

## 4. Materials and Methods

### 4.1. Materials

**Figure 1:**
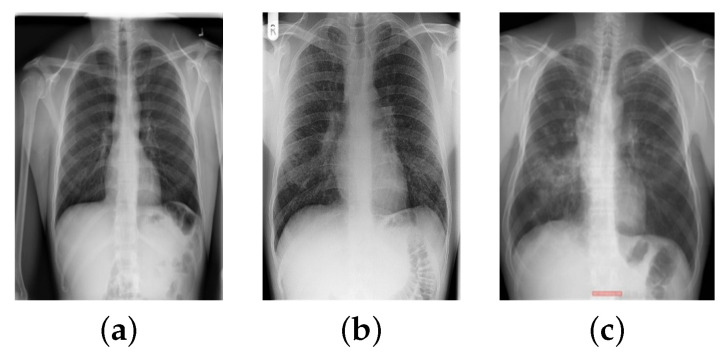
Different classes of chest X-ray images: (**a**) normal, (**b**) sarcoidosis-infected, (**c**) TB-infected.

To conduct this research, a combination of images from different sources was used. For TB and normal chest X-ray images, four publicly available datasets were examined. These datasets include Kaggle Chest X-rays and the accompanying lung mask dataset [38], the National Library of Medicine (NLM) dataset [39], the Belarus dataset [40], and the RSNA pneumonia detection challenge dataset [39]. For sarcoidosis, 231 datasets were obtained from six different national hospitals in Egypt. Table 1 shows the data collection sources for each class in tabular form. Figure 1 illustrates a selection of chest X-ray images and their classifications. In Figure 1b,c, it can be seen how difficult it is to visually distinguish sarcoidosis from TB.

### 4.2. Background

#### 4.2.1. Training Convolutional Neural Networks (CNNs)

The input is routed across the network layers during the forward phase when training a CNN [19,41,42]. Gradients are back-propagated and neuron weights are updated during the backward phase. Layer *l*’s neuron I receives input from layer *l*-1’s neuron *j* in a forward pass computed as in Equation (1). The output is calculated using the ReLu function as in Equation (2).

(1) $$In_{i}^{l}=\sum_{j=1}^{n}W_{ij}^{l}x_{j}+b_{i}$$

(2) $$out_{i}^{l}=m\left(0,In_{i}^{l}\right)$$

A Conv layer, a max-pooling layer, and a SoftMax layer comprise the CNN’s three layers. The pooling layer slides the feature map (NN) into a square window (KK), and then picks the highest or average value of the features within the sliding. The classification probability of each class type is calculated in the final layer using the activation SoftMax function as in Equation (3).

(3) $$out_{i}^{l}=\frac{e^{\ln_{i}^{l}}}{\sum_{i}e^{out_{k}^{l}}}$$

During the back-propagation phase, CNNs are trained by lowering the cost function in Equation (4).

(4) $$C=-\frac{1}{m}\sum_{j=i}^{m}\ln p(y^{j}|X^{j})$$

where:

*m*: the number of samples in the training dataset.
$X^{j}$: the $i^{th}$ sample with the label $y^{j}$ in the training dataset.
$p(y^{j}|X^{j})$: the probability of correct classification.

The cost function C is minimized using the stochastic gradient descent optimization technique, and the training cost is calculated using the mini-batch cost. Then, in the next iteration, weights are modified using the equations below:

(5) $$\gamma^{t}=\gamma^{\frac{tN}{m}}$$

(6) $$V_{l}^{t+1}=\mu V_{l}^{t}-\gamma^{t}\alpha_{l}\frac{\partial C}{\partial W_{l}}$$

(7) $$W_{l}^{t+1}=W_{l}^{t}+V_{l}^{t+1}$$

where:

$W_{l}^{t}$: the weights for layer *l* at iteration *t*
*C*: the cost of the mini-batch
α: the learning rate
$\alpha_{l}$: the layer *l* learning rate
γ: the rate of scheduling
μ: the impact of lastly updated weights of neurons in the recent iteration.

#### 4.2.2. Transfer-Learning-Based Convolutional Neural Network

The neuronal weights of the CNN layers are updated using Equation (6) after each epoch during the training phase. For training and tuning, deep networks require a large dataset. However, for small datasets, locating the local minima for the cost function in Equation (5) is challenging, leading to the network being over-fitted. As a result, the pre-trained model was used to establish the weights. The pre-trained models used in this research are described below.

##### EfficientNet

**Figure 2:**
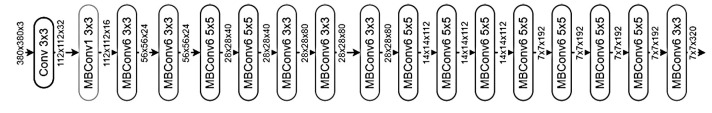
Schematic illustration of EfficientNet-B0 architecture.

By scaling down the model equally in all three dimensions, namely depth, width, and resolution, EfficientNet [43] achieves better results. There are seven models between B0 and B6 ; the number of parameters does not increase, but the model’s accuracy does. EfficientNet B0, the model from which all subsequent EfficientNet models are formed, is depicted schematically in Figure 2.

Depth, breadth, and resolution are the scaling dimensions of a Convolutional Neural Network (CNN). The number of layers within a network determines its depth, which is equivalent to its breadth. This is only a representation of the CNN’s network. The image resolution delivered to the CNN is referred to as resolution. EfficientNet uses a simple and effective scaling technique that utilizes a compound coefficient to equally scale network depth, breadth, and resolution, as Equations (8), (9) and (10) respectively.

(8) $$\mathrm{depth}\to d=\alpha^{\varphi },\;\alpha \geq 1,$$

(9) $$\mathrm{width}\to w=\beta^{\varphi },\;\beta \geq 1;$$

(10) $$\mathrm{resolution}\to r=\gamma^{\varphi },\;\gamma \geq 1;$$

where α, β, and γ: are constants and determined using the aforementioned technique.

We use φ to denote a user-specified coefficient that determines how many resources are accessible, whereas α, β, and γ determine how these resources are allocated to network depth, width, and resolution, respectively. The compound scaling approach scales the baseline EfficientNet-B0 in two stages:

1. Set φ = 1, assuming that twice as many resources are available, and apply a grid search for α, β, and γ.
2. Set α, β, and γ as constants according to the values determined in the previous step and investigate with different values of φ. The different values of φ produce EfficientNets B0–B6. Table 2 shows the input sizes and the number of total parameters for each EfficientNet model.

##### VGG16

**Figure 3:**
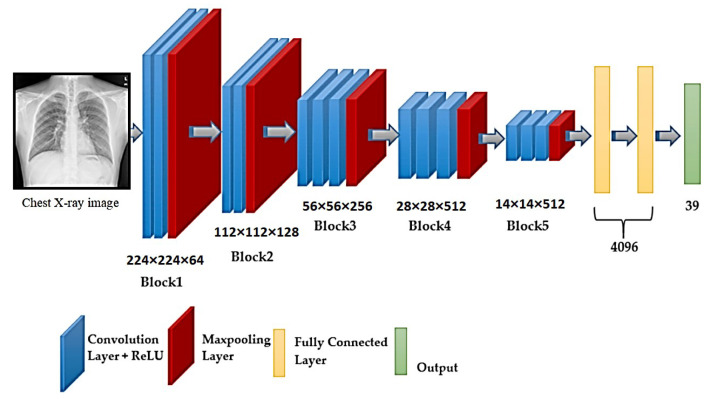
Schematic illustration of VGG16 architecture.

The VGG16 [44] design is based on the convolution and maximum pooling layers layout (Figure 3). There are three FC layers, with ReLU activating the first two and Softmax activating the third. The input layer may receive images with a size of 224 × 224 pixels, and the design contains 16 layers and 138 million parameters.

##### AlexNet

**Figure 4:**
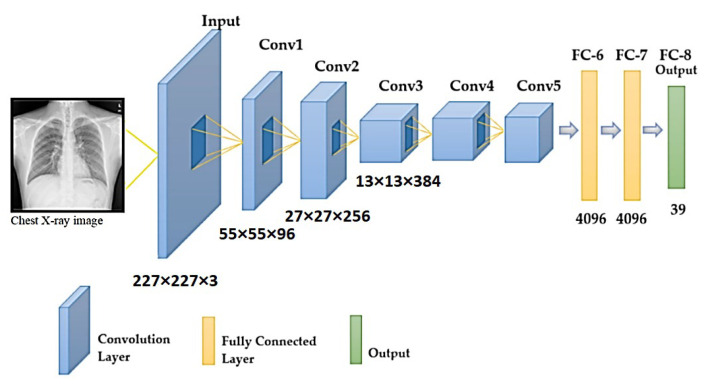
Schematic illustration of the AlexNet architecture.

The AlexNet [45] model, which uses an 8-layer CNN architecture, has 61 million parameters. There are five convolutional layers in the AlexNet architecture (Figure 4), three fully connected layers, and finally, the Softmax layer, which requires an image with a resolution of 227 × 227 for input. The ReLU activation function is used in the convolutional and fully connected layers of this system. The fully connected layer (FC-8) of the AlexNet architecture is linked to the Softmax layer through 39 neurons. The output value of the Softmax layer is the ratio of the input image to the output, which is represented by the output.

##### ResNet50

**Figure 5:**
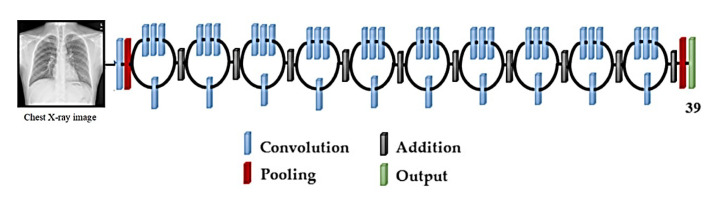
Schematic illustration of the ResNet50 architecture.

The ResNet50 [46] architecture was designed to overcome difficulties such as non-linear layers, identity mappings that do not learn, and deterioration. A network made up of residual unit stacks is known as ResNet50 (Figure 5). Residual units are used as building components to construct the network. Convolution and pooling layers are used to create these units. The input images have a resolution of 224 × 224 pixels, and the design includes 3 × 3 filters.

##### Inception V3

**Figure 6:**
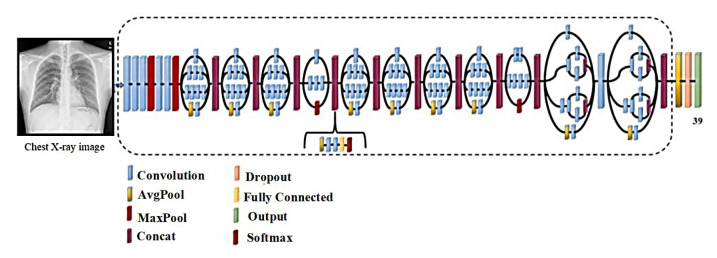
Schematic illustration of the Inception V3 architecture.

The model includes symmetrical and asymmetrical building elements, such as convolutions, average pooling, maximum pooling, dropouts, and entirely connected layers (Figure 6). The Softmax function founded in the last layer of Inception is contained in V3 architecture [47]. This architecture comprises 42 layers, with the input layer receiving information at a resolution of 299 × 299 pixels.

### 4.3. Methods

#### 4.3.1. Overview

**Figure 7:**
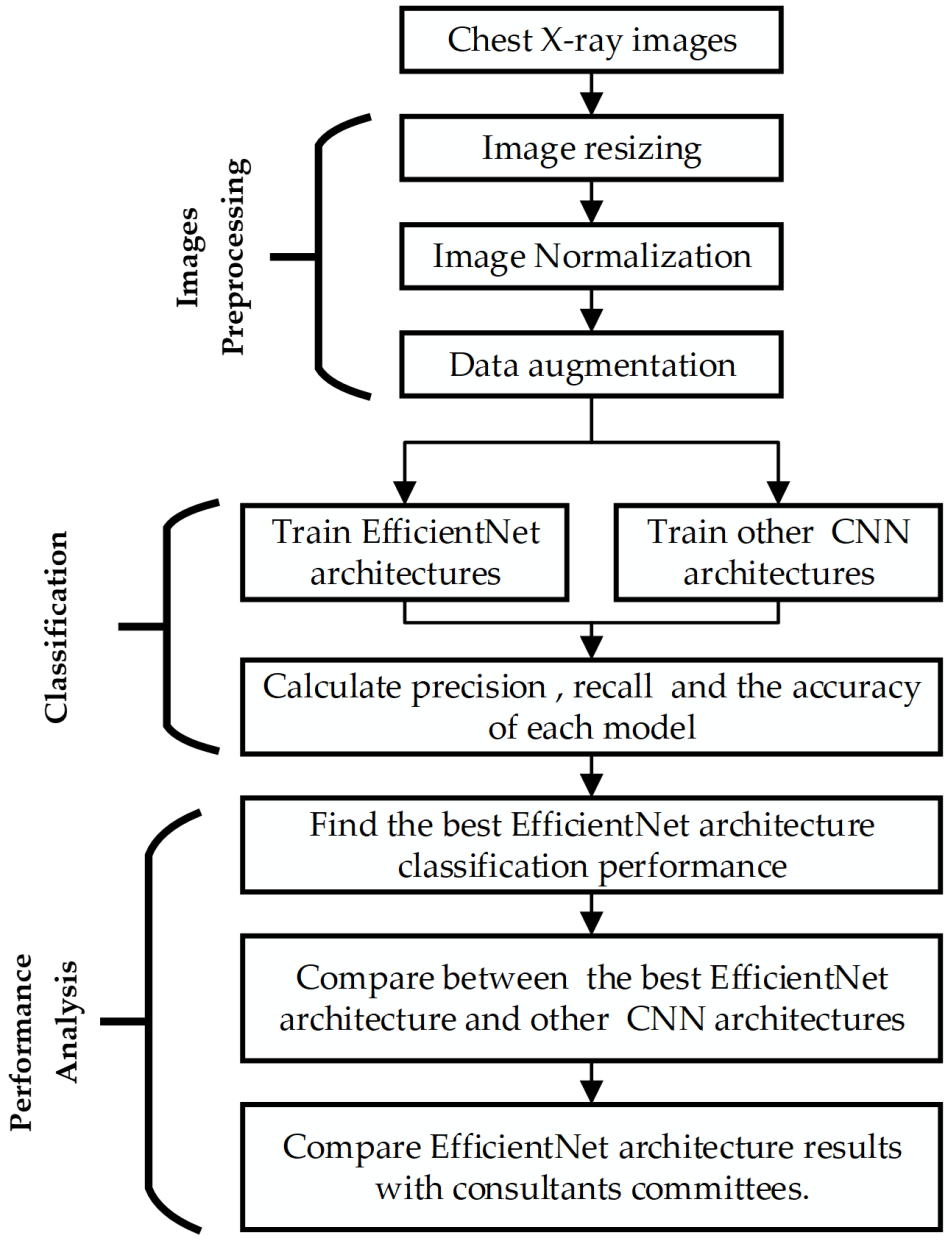
The general methodology for X-ray image classification employing a deep learning approach.

Figure 7 is a schematic diagram of the overall methods used in this study. The approach is divided into three primary stages, including (1) chest X-ray image pre-processing, (2) classification, and (3) classifier performance analysis and evaluation. Each step is explained below.

#### 4.3.2. Pre-Processing

To improve the classification results, some pre-processing steps were performed. The tasks completed during pre-processing are described below.

- Image Resizing: The X-ray image datasets for this study were obtained from multiple sources; therefore, the images were of various sizes. Each network only accepts a specific size of image. Each image was reduced to the specific sizes required by the networks while keeping its important features. The images were resized according to the recommended input size of each EfficientNet architecture, as shown in Table 2.
- Image Normalization: Different manufacturers of X-ray devices may provide different-looking X-ray images for the same patient. Overfitting to the device pixel distributions is quite a big problem in computer-aided diagnostic devices; therefore, it is standard practice to apply contrast normalization to minimize this problem. The general idea is to unify the distribution of pixels. This makes X-rays appear a little darker. This procedure generates a view that radiologists would not see in their standard workplace. Using the Reinhard and Macenko approaches, X-ray images were stain-normalized [43,48,49]. A reduction in the color discrepancies of X-ray images improves the classification accuracy of EfficientNet models.
- Data Augmentation: The normalized X-ray images were augmented before introduction into the EfficientNet model for training. The process of increasing the number of original images in a collection is known as data augmentation [38,50]. This strategy helps to eliminate the overfitting problem that arises when a model learns enough from the training data but cannot classify images of undetected X-rays. Table 3 illustrates the augmentation settings used on the stain-normalized X-ray images. In this study, the number of normal images was 1010, which is twice as many as the number of TB- and sarcoidosis-infected images. Therefore, the TB-infected images were augmented from 563 to 1126, and the sarcoidosis-infected images were increased from 231 to 462.

#### 4.3.3. Classification Stage

Seven EfficientNet architectures between B0 and B6 achieved a classification model for analyzing chest X-ray images to distinguish between normal, TB-infected and sarcoidosis-infected cases. The suggested EfficientNet architecture’s results were also compared to the state-of-the-art CNN architectures, such as AlexNet, ResNet50, VGG16, and Inception V3. All deep learning models were trained in Google Colab with GPU support. All code was written in Python 3.10.1 using Keras version 2.7.0. Keras is an open-source deep learning framework.

#### 4.3.4. Evaluation of the Classification Performance of the Proposed Methodology

The proposed methodology was evaluated in two stages. The first stage employed sensitivity, precision, and accuracy measures to determine the optimum EfficientNet architecture. The formulas used in calculating these measures were as Equations (11), (12) and (13) respectively.

(11) $$Sensitivity=\frac{TP}{TP+FN}\times 100\%$$

(12) $$Precision=\frac{TP}{TP+FP}\times 100\%$$

(13) $$Accuracy=\frac{TP+TN}{TP+TN+FP+FN}\times 100\%$$

where TP (true positive) and TN (true negative) represent the number of correctly classified chest images that belonged to the normal and infected classes, respectively. In addition, FN (false negative) and FP (false positive) represent the number of misclassified X-ray images in the normal and infected classes, respectively [51].

In the second stage, the results of the best EfficientNet model were compared to the consultant committees’ classification results. Three consultant committees were constituted from three separate Egyptian respiratory disease hospitals (Cairo University Hospital (Kasralainy Hospital), Kobry El-kobba Military Hospital, and El-Abaseya Hospital). Each team was comprised of three specialized physicians (one specialized in critical care units and two specialized in respiratory diseases and allergies). All physicians had at least 15 years of experience. Images of ten patients were selected randomly from our datasets: two normal, three infected with TB, and five sarcoidosis-infected cases. The ten X-ray images were presented to the consultant committees. The committees’ decisions were compared to the classifier results.

## 5. Results and Discussion

The primary goal of this study is to obtain the best classification technique that classifies normal, sarcoidosis-infected, and TB-infected images with the highest precision to help physicians distinguish sarcoidosis from TB using X-ray images. To achieve this goal, the classification abilities of the EfficientNet deep learning architecture were evaluated with respect to chest X-ray images, and we compared the EfficientNet architecture’s performance to that of the most recent CNN models in the literature. All deep learning architectures used in this study were trained via transfer learning, as stated in Section 4.2.2.

**Figure 8:**
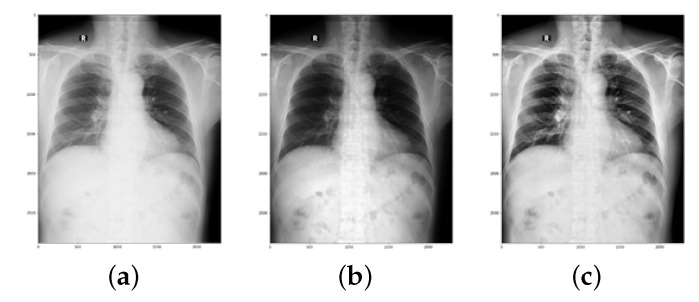
Stain-normalization outcomes: (**a**) original image, (**b**) Reinhard approach, and (**c**) Macenko approach.

To acquire a better understanding of the impact of Reinhard’s and Macenko’s normalization approaches on the classification performance of the deep learning architecture, all X-ray images were stain-normalized by these algorithms in the first stage. In addition, investigations with non-normalized images were carried out. The outcomes of the stain-normalization approaches employed in this study are shown in Figure 8.

**Figure 9:**
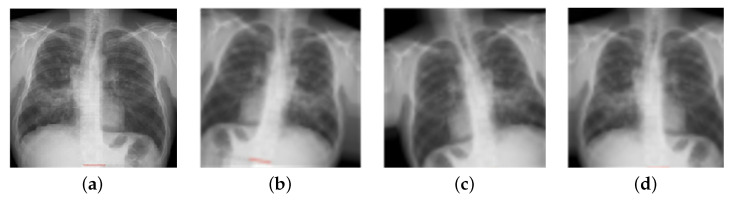
Images of chest X-rays after using random data augmentation techniques: (**a**) original, (**b**) after clockwise rotation by 10 degrees, (**c**) after anti-clockwise rotation by 10 degrees, and (**d**) after 10% translation.

The second stage of this research involved data augmentation; there were 1010 normal samples in the datasets. This value was almost four times higher than the number of sarcoidosis-infected images and 1.5 times higher than the number of TB-infected images. As a result, it was essential to augment the dataset symmetry for the sarcoidosis- and TB-infected images. Furthermore, studies [48,50] reveal that data augmentation obtains new datasets and increases the classification accuracy of deep learning systems by enriching the original datasets. As illustrated in Figure 9, two image augmentation techniques (rotation and translation) were used to generate additional X-ray images of sarcoidosis- and TB-infected lungs. Augmentation techniques were adopted to ensure that X-ray images were not unduly distorted and to prevent the loss of important image features. Images were turned clockwise and counterclockwise to achieve image augmentation (images were rotated with an angle of 5 and 10 degrees in each direction). Images were translated by shifting them vertically (height shift), horizontally (width shift), or both vertically and horizontally (images were translated by 10% and 15%).

In the third stage, we investigated whether normalization approaches could help classification models be more accurate. For sarcoidosis, TB, and normal X-ray classification, seven EfficientNet architectures were trained on non-normalized and normalized X-ray images. With 10-fold cross-validation, the X-ray images were separated into two groups: 80% for training and 20% for testing. The hyper-parameter settings for the EfficientNet models during the training phase are as outlined in Table 4.

**Figure 10:**
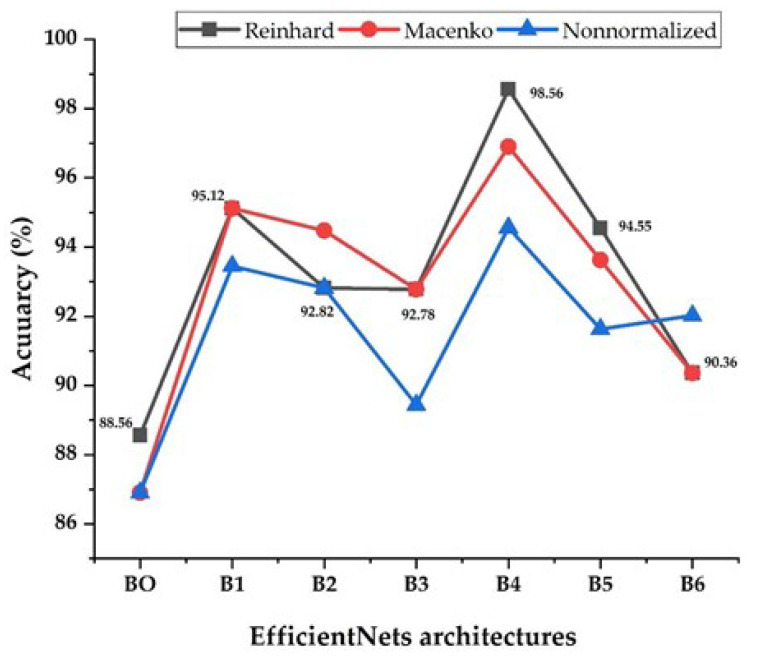
Performance accuracy for the EfficientNet architectures with non-stain-normalized and stain-normalized images.

Figure 10 shows each EfficientNet architecture performance in terms of its accuracy using stain-normalization techniques and non-normalized images. In general, the EfficientNet models performed better on normalized X-ray images than on non-normalized images, according to the results of the experiments. It’s worth noting that the average gain in accuracy for EfficientNets-1, B2, B3, B4, B5, and B6 appears to have shifted slightly. While EfficientNet-B4 saw a consistent increase, the larger EfficientNet models 5 and B6 saw a slight decrease. The EfficientNet-B0 model has the smallest number of parameters, and it performs poorly and has the lowest accuracy. This may be due to the fact that EfficientNet-B0 uses a very small input size. The image structures may be affected by resizing the X-ray images to 224 × 224, preventing the model from extracting the features. This could be due to over-parameterization, as opposed to the larger dataset size for the larger EfficientNet models, which did not appear to perform as well. For EfficientNets-B4 and B5, the Reinhard approach outperforms the Macenko approach. For EfficientNet-B4, the Macenko method and non-normalized images scored best. The Macenko and Reinhard approaches had the same results for EfficientNets-B1, B3, and B6.

In comparison to the other six models, the EfficientNet-B4 model achieved outstanding results. This model’s accuracy (98.56%, 96.9%, and 94.56% for Reinhard, Macenko, and non-normalized images, respectively) is impressive. The results show that this approach was the most effective at learning and identifying important features from training data. This approach also has the advantage of being simpler and having fewer trainable parameters than previous EfficientNets B5 and B6, implying faster training.

Classification accuracy alone can be deceiving. The sensitivity and precision rates, which are shown in Table 5, were calculated as a consequence. The sensitivity of the EfficientNet-B4 model is consistently high across all models. The best sensitivity (98.36%) and precision (98.67%) for this model came from using images normalized with the Reinhard approach. The Macenko approach achieved close results with a sensitivity of 96.9% and a precision of 97.11%.

While training the images adjusted with the Reinhard and Macenko methods, the EfficientNets-B1 and B2 generated comparable results, with sensitivities and precisions of around 95.00% and 93.00%, respectively. Even though EfficientNets B1 and B2 have different numbers of parameters, they both accept the same input size. For extracting essential features from X-ray images in the database, input sizes of 240 × 240 and 260 × 260 are sufficient. This comparison clearly shows that EfficientNet-B4 outperforms all other EfficientNet models.

**Figure 11:**
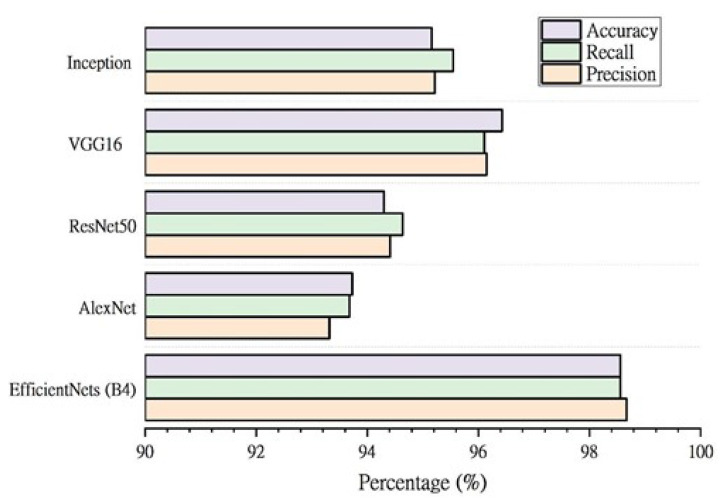
Comparison between the EfficientNet-B4 model and the state-of-the-art approaches.

To further evaluate the performance of the EfficientNet models, the results were compared to similar deep learning approaches. The classification performance of pre-trained designs employed in earlier approaches is shown in Figure 11. This demonstrates that the EfficientNet-B4 model produces superior accuracy (98.56%), precision (98.67%), and sensitivity (98.56%) for distinguishing between chest X-ray images for normal, TB-infected and sarcoidosis-infected cases. Furthermore, when compared to all comparable models, EfficientNet-B4 has the fewest parameters (about 17.9 million) and hence is computationally cheaper than the others due to its lightweight nature. It also has the shortest training time per epoch.

For further evaluation of the proposed approach, it was compared to conventional diagnostic tests that are reported in several research studies [52,53,54,55,56,57,58,59,60,61,62,63], as shown in Table 6. From the table, it can be seen that sarcoidosis and tuberculosis are disorders that closely resemble each other. For distinguishing sarcoidosis from TB, physicians usually employ physical examinations, biopsy tissue, blood analysis, urine tests, and a tuberculin skin test; this approach is painful for patients, time-consuming, expensive, and uses techniques prone to human error. In contrast, the proposed approach requires only a chest X-ray image to differentiate between TB and sarcoidosis.

Finally, the results of the proposed system were compared to the assessments of the consultant committees for the same 10 patients categorized by the recommended system. The number of patients in each class (normal, tuberculosis-infected, and sarcoidosis-infected), as well as the number of accurate EfficientNet-B4 and consultant committee classifications, are shown in Table 7.

All normal chest X-ray images were correctly diagnosed by the EfficientNet-B4 and consultant committees. EfficientNet-B4 misclassified one TB image, resulting in a lower number of inaccurate predictions than the consultant committees, which were unable to diagnose two out of three TB cases. Although the consultant committees failed to diagnose all five cases of sarcoidosis (0% true prediction), the proposed method correctly identified three of the five cases of sarcoidosis. The advisory committees in our study attributed their inability to diagnose sarcoidosis using X-ray images alone to the fact that physicians can recognize anomalies from X-ray images, but they require further blood tests in addition to chest swabs to distinguish sarcoidosis from tuberculosis. It could be concluded that the proposed method has a considerable benefit in detecting sarcoidosis using X-ray images without requiring the patient to undertake costly laboratory tests or lung smears, which are often painful for the patient and take a long time to obtain results. The proposed framework to detect sarcoidosis in patients using chest X-ray images may be employed clinically after more examination.

## 6. Conclusions

In this study, seven EfficientNet versions with transfer learning were utilized to classify chest X-ray images into three categories: normal, TB-infected, and sarcoidosis-infected. The EfficientNet-B4 model, which has around 17106 parameters, was the best of the seven models and achieved remarkable results, with accuracy, sensitivity, and precision rates of 98.56%, 98.36%, and 98.67%, respectively. Experiments have shown that this architecture, using X-ray images of the chest, can extract and learn global information. Additionally, the impacts of two alternative stain-normalization procedures were evaluated and compared to images that were not normalized. The B4 model had better performance when using the Reinhard technique, according to the results of this study. When compared to existing deep learning architectures (AlexNet, ResNet50, VGG16, and Inception V3) utilized to analyze chest X-ray images in the literature, as well as three special advisory committees, the proposed EfficientNet-B4 model was more successful. The results demonstrate that the EfficientNet architecture’s B4 model delivered the best outcomes. The proposed method offers a competitive improvement in terms of detecting sarcoidosis utilizing X-rays without requiring the patient to undergo expensive laboratory tests or lung smears, which are typically painful for the patient and take a long time to produce results. Based on our proposed methodology, we expect that the medical community will accept our proposed framework for classifying patients with sarcoidosis and tuberculosis using chest X-ray images.

In the future, it would be impressive to augment the capacity of the EfficientNet-B4 architecture to detect stages of pulmonary sarcoidosis and to increase the number of cases diagnosed by consultant committees for comparison. In addition, the performance of the proposed model will be investigated using MRI images. This is a challenging path worth pursuing. Finally, the suitability of the proposed model for accumulative learning will be evaluated.

## Figures and Tables

**Table 1:** Image categories and their collection sources.

Class	Source	Number of Images
Normal	Kaggle	360
	NLM database	400
	RSNA CXR dataset	250
TB	Belarus database	169
	NLM database	394
Sarcoidosis	Six national hospitals in Egypt	231

**Table 2:** Number of parameters in each EfficientNet and its corresponding input image size.

EfficientNet Model	Input Image Size	Number Parameters $\times 10^{6}$
B0	224 × 224	4.3
B1	240 × 240	6.8
B2	260 × 260	8
B3	300 × 300	11
B4	380 × 380	17.9
B5	456 × 456	28.7
B6	528 × 528	41.1

**Table 3:** The settings of the data augmentation applied to the stain-normalized X-ray images.

Augmentation Type	Value
Rescaling	According to each EfficientNet model
Rotation range	$5^{\circ }$ and $10^{\circ }$
Range of width shifts	0.1
Range of height shift	0.1

**Table 4:** The hyper-parameter setting for EfficientNet architectures.

Hyper-Parameter	Setting
Patience	5
Learning rate	0.001
Size of mini-batch	32
Optimizer	SGD
Activation function	Softmax

**Table 5:** Precision sensitivity for the EfficientNet models with the stain-normalization method.

EfficientNet Architectures	Precision		Sensitivity	
	Reinhard	Macenko	Reinhard	Macenko
B0	94.88	92.9	94.56	92.9
B1	95.16	93.69	95.82	93.48
B2	95.16	93.69	95.82	93.48
B3	93.49	93.05	92.78	92.78
B4	98.67	97.11	98.36	96.9
B5	90.74	94.1	90.63	93.97
B6	91.34	91.15	90.36	90.21

**Table 6:** Comparison between conventional diagnostic tests for sarcoidosis, tuberculosis, and the proposed approach.

Test Type	Indication for Tuberculosis	Indication for Sarcoidosis	Proposed Approach
Physical examination	Coughing for three or more weeks, coughing up blood or mucus, chest pain, weight loss, fatigue and fever [52]	Fatigue, fever, weight loss, and erythema nodosum [53]	Not required
Peripheral blood count	High lymphocyte count [54]		
Renal function tests	Unclear for tuberculosis diagnosis [55]	High level of calcium, urea, and creatinine [56]	
Urine analysis	Urine analysis currently offers little utility for the diagnosis of tuberculosis [57]	Hypercalciurea [53]	
Pulmonary function tests	Just used to indicate pulmonary involvement and disease severity, but not to determine whether TB or sarcoidosis is present [53]		
Tissue biopsy	This method is probably the most useful one for the diagnosis of bone and joint tuberculosis [58]	For the presence of granuloma (lungs, lymph node, skin, salivary gland, conjunctiva) [53]	
Bronchial biopsy	Transbronchial lung biopsy (TBLB) is a helpful examination for pulmonary tuberculosis [59]	Flexible bronchoscopy has a very high diagnostic yield in all stages of suspected sarcoidosis [53]	
Tuberculin skin test (Mantoux)	Determining whether a person is infected with mycobacterium tuberculosis [60]	Negative in most sarcoidosis patients [53]	
Electrocardiogram (ECG)	Patients with pulmonary tuberculosis often have a normal ECG [61]	Repolarization disturbances, ectopic beats, and rhythm abnormalities [62]	
MRI	MRI is the most sensitive modality for early diagnosis and follow-up of spinal TB [63]	Detect neurological involvement, spinal cord, meninges, skull vault, and pituitary lesions [53]	Not investigated to improve diagnostic accuracy
Chest X-ray	A posterior-anterior chest radiograph is used to detect chest abnormalities [53]		Required

**Table 7:** Comparison between the proposed approach and conventional diagnostic tests for sarcoidosis and tuberculosis.

Cases	Number of Actual Cases	EfficientNet-B4	Committees of Consultants
Normal	2	2 (100%)	2 (100%)
Tuberculosis	3	2 (67%)	1 (33%)
Sarcoidosis	5	3 (60%)	0 (0%)

## Data Availability

Not applicable.
